# High-Resolution X-Ray Computed Tomography: A New Workflow for the Analysis of Xylogenesis and Intra-Seasonal Wood Biomass Production

**DOI:** 10.3389/fpls.2021.698640

**Published:** 2021-08-06

**Authors:** Romain Lehnebach, Matteo Campioli, Jozica Gričar, Peter Prislan, Bertold Mariën, Hans Beeckman, Jan Van den Bulcke

**Affiliations:** ^1^UGCT–UGent-Woodlab, Laboratory of Wood Technology, Department of Environment, Faculty of Bioscience Engineering, Ghent University, Gent, Belgium; ^2^AMAP Laboratory (botany and bio-informatics of plant architecture and vegetation), Université Montpellier, CIRAD, CNRS, INRAE, IRD, Montpellier, France; ^3^Research Group PLECO (Plants and Ecosystems), Department of Biology, University of Antwerp, Antwerp, Belgium; ^4^Department of Yield and Silviculture, Slovenian Forestry Institute, Ljubljana, Slovenia; ^5^Royal Museum for Central Africa, Service of Wood Biology, Tervuren, Belgium

**Keywords:** high-resolution X-ray computed tomography, microtomy, tree growth, xylogenesis, secondary growth phenology

## Abstract

Understanding tree growth and carbon sequestration are of crucial interest to forecast the feedback of forests to climate change. To have a global understanding of the wood formation, it is necessary to develop new methodologies for xylogenesis measurements, valid across diverse wood structures and applicable to both angiosperms and gymnosperms. In this study, the authors present a new workflow to study xylogenesis using high-resolution X-ray computed tomography (HRXCT), which is generic and offers high potential for automatization. The HXRCT-based approach was benchmarked with the current classical approach (microtomy) on three tree species with contrasted wood anatomy (*Pinus nigra, Fagus sylvatica*, and *Quercus robur*). HRXCT proved to estimate the relevant xylogenesis parameters (timing, duration, and growth rates) across species with high accuracy. HRXCT showed to be an efficient avenue to investigate tree xylogenesis for a wide range of wood anatomies, structures, and species. HRXCT also showed its potential to provide quantification of intra-annual dynamics of biomass production through high-resolution 3D mapping of wood biomass within the forming growth ring.

## Introduction

Forests represent the main CO_2_ sink of terrestrial ecosystems. Understanding tree growth and carbon sequestration is of crucial interest to forecast the responses of forests to climate change and their climate mitigation potential. The forest carbon sink can be assessed with direct measurement of tree growth (i.e., Hubau et al., 2020) or indirectly through Dynamic Global Vegetation Models (DGVM). In DGVM, tree growth is often assumed to be limited primarily by photosynthesis (i.e., gross primary production) (Fatichi et al., 2019). However, experimental evidence shows that tree growth does not directly depend on the photosynthesis and that tree growth is more sensitive to environmental conditions than the photosynthesis (Fatichi et al., 2014). Therefore, more attention should be devoted to determine the direct constraints on growth exerted by the environmental conditions at different temporal scales. One way to do this is by studying wood formation (i.e., xylogenesis) that defines seasonal tree growth and carbon sequestration (Friend et al., 2019).

Xylogenesis is driven by the cambium, that is, a layer of meristematic cells, forming a cylinder along the different axes of the plant body. For temperate species, when growing conditions become favorable in spring, the cambium cells start to divide and the newly formed cells undergo different structural changes (i.e., differentiation). Xylogenesis can be divided into five sequential processes: (1) cell division and (2) cell enlargement, which drive the growth in size; (3) cell-wall thickening and (4) cell-wall lignification, which are mainly responsible for the increase in mass and C sequestration (Cuny et al., 2015); and (5) programmed cell death, which allows the woody cell skeleton to be used as mechanical support and conduit for water transport. The wood formation and its dynamics are strongly influenced by environmental conditions, such as the temperature, day length, and water availability (Delpierre et al., 2016; Dox et al., 2021). Therefore, climate change is expected to affect the tree growth and carbon accumulation through the modulation of the timing and duration of xylogenesis.

An appealing method to study the phenology and the seasonal dynamics of wood formation as well as the climate-wood growth relationships consists in monitoring wood development in small cores (i.e., microcores) collected at regular time intervals during the growing season (Rossi et al., 2006). Seasonal observations of anatomical features characterizing the wood formation (e.g., cell morphology, cell developmental stage, or number of cells in a given stage) are made possible by brightfield microscopy after histological slicing and staining. Most of the xylogenesis studies and the development of the histological approach (referred hereafter as “microtomy”) have been performed on conifers with a homogeneous wood structure (Rossi et al., 2009, Deslauriers et al., 2008, Camarero et al., 2010). This intensive work on conifers also permitted to refine the understanding of the asynchrony between xylem size increase and biomass production through a detailed quantification of cell development kinetic during xylogenesis (Cuny et al., 2015). This approach allowed to develop a clear view on xylogenesis in conifer species (Rathgeber, 2017), while its knowledge on angiosperm species remains limited (ref. Delpierre et al., 2016, but see Cufar et al., 2008; Gričar et al., 2018; Prislan et al., 2018; Dox et al., 2020). In fact, the structural heterogeneity of angiosperm wood challenges the acquisition of xylogenesis data from a methodological point of view. Indeed, the diversity of cell types encountered in angiosperm wood (vessels, fibers, and parenchyma) makes cell-counting and morphometrics difficult, while limiting the acquisition of information on the xylogenesis dynamics. Therefore, even if some authors have been able to quantify the proportion of growth increment attributed to the different stages of development (Prislan et al., 2018), intra-seasonal studies of secondary growth in angiosperms have been often limited to the estimation of the timing of qualitative events (e.g., earlywood vessel formation, latewood formation). From the biomass accounting point of view, bypassing cell-level information by characterizing the biomass accumulated during the season at the tissue level is a promising perspective. In this respect, the methodology of Andrianantenaina et al. (2019), based on an estimation of the apparent density of the forming wood, should allow to overcome the issue of structurally heterogeneous wood and thus to quantify the dynamics of intra-annual biomass production in angiosperms. The global predominance of angiosperms stresses the importance of further methodological development allowing straightforward xylogenesis measurements across the diversity of wood structures and species found within the angiosperm group, and across the different climates and biomes.

Investigating how tree growth interacts with environmental factors at a global scale requires the development of a high-throughput methodology for xylogenesis studies applicable also to angiosperms. Such methodology should avoid the histological preparation and analysis that are labor intensive, requiring specific expertise, and several manual steps, such as paraffin embedding, slicing, staining, mounting, and observation using bright field microscopy. So far, the labor intensity of the manual steps during sample preparation (requiring skilled technicians) and data collection (observations, requiring skilled wood anatomists) has limited the automation of the microtomic approach, pointing out the importance to develop an automated methodology to study seasonal wood formation.

High-resolution X-ray computed tomography (HXRCT) is becoming popular in the analysis and imaging of plant anatomy (Brodersen and Roddy, 2016). Different research fields related to tree growth and functioning, such as dendrochronology (De Mil et al., 2016; Van den Bulcke et al., 2019) and ecophysiology (Knipfer et al., 2015; De Baerdemaeker et al., 2019), seized the opportunity of HXRCT for substantial methodological improvements. However, HXRCT has never been used in the demanding field of xylogenesis. Producing three-dimensional gray scale images with pixel values (attenuation coefficients) directly related to the density of the material, HXRCT represents a powerful mean to study xylogenesis. More precisely, HXRCT can be used to track cell wall density change occurring during the formation and thickening of the secondary cell wall in order to differentiate the mature wood from the forming wood and to assess secondary growth phenology. In addition, HRXCT can quantify the biomass accumulated by trees throughout the course of the season.

The use of HXRCT in the framework of xylogenesis requires both high contrast and resolution to discern the forming cells and the cell wall. Obtaining a sufficient contrast between cell lumens and cell walls with HRXCT requires water removal from the sample. For mature xylem, water is generally removed by oven-drying the sample prior to scanning (De Mil et al., 2016). However, drying without collapsing and destroying the soft tissue, such as cambial and newly forming xylem cells, cannot be achieved through oven- or air-drying (Stuppy et al., 2003; Leroux et al., 2009).

Therefore, monitoring of xylogenesis with HXRCT requires the development of an entire workflow including sample preparation, image acquisition, and an analysis protocol, alongside a methodology to follow the cell wall density change within the forming growth ring.

**Figure 1:**
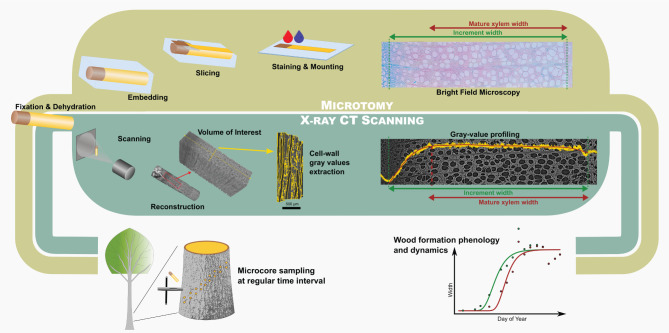
Comparison between the microtomy and HXRCT workflow developed in the present study. The flowchart covers the different steps from field sampling to data analysis through sample preparation and data extraction.

Here, a new methodology is presented and validated to study xylogenesis using HXRCT based on a new workflow to monitor cell wall density changes occurring during xylogenesis (Figure 1), applied to three tree species (black pine, common beech, and pedunculate oak) with contrasted wood anatomy growing in a temperate region. Several relevant parameters, such as the timing and duration of size growth, mature xylem production, and overall xylogenesis, as well as rates of size growth and mature xylem production, were estimated with HXRCT and validated with the classical microtomy approach. In addition, the gray scale images produced by HRXCT were used to account for seasonal biomass production dynamics. Concerning the latter aspect, the authors ask whether the intra-seasonal stem biomass production and size growth differ between conifers and angiosperms, specifically among pine, beech, and oak. While information on intra-seasonal biomass production exist for conifers (Cuny et al., 2015; Andrianantenaina et al., 2019) to the best of our knowledge, no study has investigated the intra-seasonal biomass production in angiosperms.

## Materials and Methods

### Selected Species, Study Sites, and Sampling

To assess the applicability of the HXRCT method on tree species with different functional strategies, three species growing in Belgium were selected: black pine (*Pinus nigra*), common beech (*Fagus sylvatica*), and pedunculate oak (*Quercus robur*). With 778 mm.year^−1^ of precipitation and an average monthly temperature ranging from +3°C to 17°C (average annual temperature of 10.1°C), Belgium is characterized by a maritime temperate climate (Campioli et al., 2012). Beech and oak trees were sampled in the “park of Brasschaat” (51°12′ N−4°26′ E) while pine trees were sampled in the military domain “Klein Schietveld” (51°21′ N−4°37′ E) (Dox et al., 2020; Marchand et al., 2020). The sites are at a distance of 9.5 km in a lowland (18–22 m a.s.l.) region with a similar climate. The three species present contrasted xylem anatomy: beech and oak are diffuse and ring porous species respectively, whereas black pine has the typical conifer wood structure made mostly of tracheids. Four healthy and (co)dominant trees per species were selected. From the beginning of April to mid-November 2019, two microcores were collected on each tree every two weeks using a Trephor tool resulting in 18 sampling dates. The sampling was performed between 1.3 m and 2 m above the ground following an upward spiraling pattern with a distance of 4 cm between consecutive sampling points to avoid wound reactions. The microcores were immediately transferred to an Eppendorf tube filled with 70% ethanol solution.

### X-Ray CT Workflow

#### Dehydration, Critical Point Drying, and Sample Mounting

After dehydration by increasing ethanol concentrations, the samples for HXRCT were dried to obtain a sufficient contrast between cell lumens and cell walls. Therefore, a gentle drying method was required in order to preserve the structure of the soft, newly forming wood cells. Therefore, critical point drying (CPD), a technique commonly used for the preparation of scanning electron microscopy samples (Bray, 2000), was used, but has proven its efficiency in the drying of fresh plant structures also for CT imaging (See Supplementary Figure 1 for a comparison of an air dried and CPD processed pine microcore) (Leroux et al., 2009; Gutiérrez et al., 2018).

The samples were critically point dried with liquid CO_2_ using a CPD device (Baltec CPD 030). Each drying cycle consisted of a mixing-draining procedure (repeated 10 times) allowing the gradual replacement of the dehydration media (i.e., 100% ethanol) by liquid CO_2_. Then, the samples were dried by removing the CO_2_ in a supercritical state (> 31°C, > 73 bar).

Six samples were dried at the same time. After drying, the samples were mounted on a cylindrical carbon stick (5 cm x2 mm) for scanning. The mounted samples were stabilized in the scanner room for 12 h, at 25°C and a relative humidity of 40%, to avoid sample movement during scanning.

### X-Ray CT Scanning and Reconstruction

Samples were scanned with the Nanowood X-ray CT scanner, developed at the Ghent University Centre for X-ray tomography (UGCT; http://www.ugct.ugent.be) (Dierick et al., 2014). The directional X-ray tube (Microfocus, Hamamatsu) was operated at a tube voltage of 70 kV and a target current of 100 μA. A large (25 x 20 cm, 1920 x 1536 px) Varian Si flat detector was used allowing to capture the region of interest (i.e., innermost phloem layers, cambium, and forming xylem growth ring) in a single field of view. A total of 2001 projections were taken with an exposure time of 700 ms per projection, resulting in 25 min per scan.

Scanned volumes were reconstructed with the Octopus reconstruction software (Vlassenbroeck et al., 2007), including Paganin phase contrast filtering (Paganin et al., 2002). The reconstructed volume, with an approximate voxel pitch of 2.49 μm, consisted of 16-bit tangential images running from the bark to the inner side of the xylem.

### Processing and Analysis of the Reconstructed Volume

#### Selection of the Regions of Interest and Gray-Value Extraction

When sampling, the axis of the Trephor tool can substantially deviate from both the radial direction and the transverse plane of the stem. Therefore, the reconstructed volume was reoriented to realign the cambium surface parallel to the volume edge in both transverse and longitudinal plane (Supplementary Figure 2) using ImageJ (Rasband, 2012). A volume of interest (VOI) containing the inner layers of the phloem, the cambium, and the forming xylem growth ring was selected while avoiding selection of wide rays, resin ducts, or cracks.

This VOI was then processed with Octopus analysis, formerly distributed by the UGCT spin-off company XRE (now TESCAN-XRE, part of the TESCAN ORSAY HOLDING a.s.) (Brabant et al., 2011). For each tangential slice of the VOI, a first thresholding was applied to create a mask covering the area containing the sample. The masks were used to create a first region of interest (ROI) image stack containing xylem pixels (xylem ROI stack). For each tangential slice of the xylem ROI stack, a second thresholding was applied to create a mask excluding the pixels associated to cell lumens. The masks were used to create a second region of interest image stack containing cell wall pixels exclusively (cell wall ROI stack). Both xylem and cell wall ROI stacks of each microcore were then processed with R software (R Development Core Team, 2019), using the packages “*magick”* (Ooms, 2020) and “*imager”* (Barthelme, 2019), to extract the mean gray value per ROI resulting in two radial gray-value profiles per microcore, herein after referred to as “xylem gray profile” and “cell wall gray profile,” respectively.

#### Cell Wall Gray-Value Profiling: Rationale

**Figure 2:**
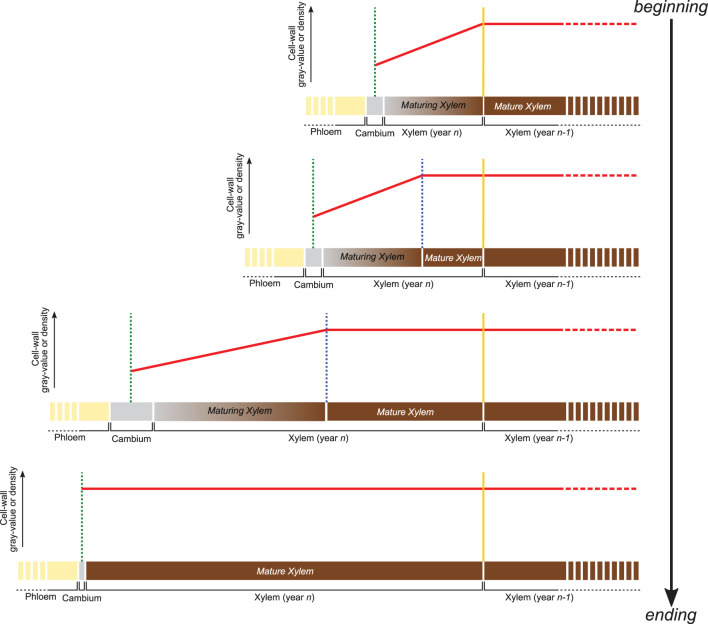
Variation of cell wall gray value or density within the forming growth ring. Following cell wall maturation during xylogenesis, the cell wall density (red line) is expected to increase from the cambium (green dotted line) to the most recent mature xylem layer (blue dotted line). Within the mature xylem, the cell wall is fully developed and its basic density is assumed to be constant until the growth ring boundary (yellow dotted line) and beyond. The distance from the cambium at which the phase transition occurs, allows estimating the width of both mature and maturing xylem.

The gray-value profiling methodology developed here relies on the positive and linear relationship between the gray value of HXRCT-derived images and the density of the scanned material. Therefore, cell wall density changes occurring within the forming growth ring can be assessed through cell wall gray value changes. From a structural point of view, the forming growth ring consists of a maturing xylem zone (where xylogenesis occurs), and a mature xylem zone, where xylogenesis already ended (Figure 2). Within the maturing xylem, the secondary cell wall is formed and lignin is deposited resulting in an increase of the cell wall density from the cambium until the boundary with the mature xylem zone.

Apart from secondary changes occurring many years later, such as heartwood formation, the structure of the fully mature cell wall does not change in time (Lehnebach et al., 2019), its density is assumed to vary both between and within species in a very narrow range (1.4–1.5 g/cm3). Cell wall density, and consequently cell wall gray value are therefore assumed to remain constant within the mature xylem zone. Consequently, the cell wall density profile along the growing ring follows a two-phased pattern with (1) an increase from cambium to the end of the maturing xylem zone followed by (2) a constant density toward the innermost layers of the mature xylem zone.

Identifying the distance from the cambium at which the phase transition occurs, allows estimating the width of both maturing and mature xylem. An analytical approach is developed in this study, aiming to quantify the width of both maturing and mature xylem.

#### Cell Wall Gray-Value Profiling: Implementation

**Figure 3:**
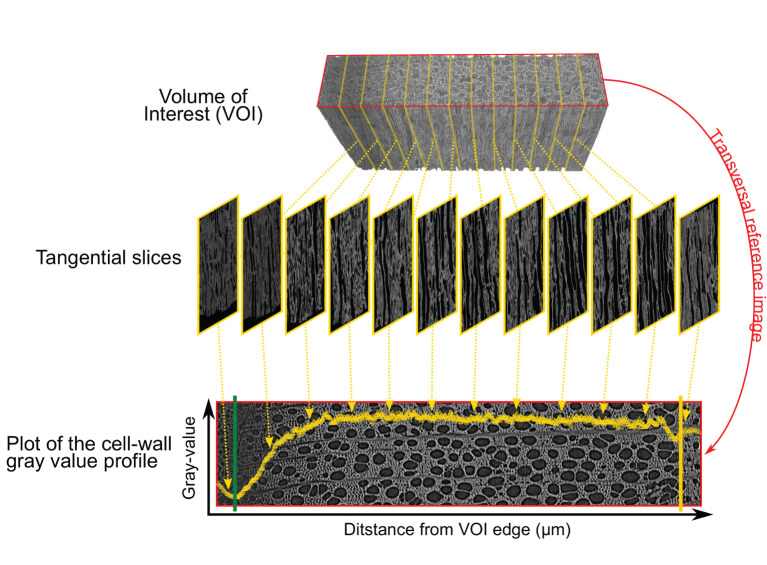
Plot of the cell wall gray profile. The volume of interest (VOI) containing the inner layers of the phloem, the cambium, and the forming xylem growth ring (top) is sliced tangentially (middle). Averaging the cell wall gray values of each tangential slices results in a cell wall gray value profile plotted over the transversal reference image (bottom). The reference image is in the red frame (top and bottom image), the cambium and previous ring boundary are highlighted by the green and yellow lines respectively.

For each sample, the cell wall gray profile was plotted over a transversal reference image, with each data point corresponding to the average gray value of the corresponding tangential slice (Figure 3). We used the smoothing property of a generalized additive model (GAM) to analyze the cell wall gray profile of each microcore. GAMs are extremely versatile in fitting non-linear and non-monotonic data series, and offer the possibility to estimate and analyze their derivative. GAMs are therefore well-suited to analyze cell wall gray profile of diverse tree species. All GAMs were fitted with a Gaussian distribution and identity link function. The goodness of each fit was assessed visually.

**Figure 4:**
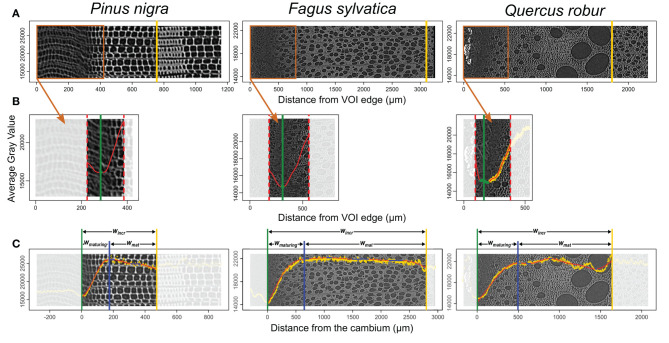
Implementation of the gray-value profiling. **(A)** The average gray value of each tangential slice is plotted on the reference cross-section image. At this stage, the previous growth ring boundary is identified (yellow vertical line). **(B)** Within the subset containing the cambium, a GAM fit (red) and its derivative allow automatic identification of the cambium position (green vertical line). **(C)** Within the growth ring, a GAM fit (red) and its derivative allows the detection of a constant gray value (blue filled circles). The constant gray value closest to the cambium is set as the boundary between maturing and mature xylem (blue vertical line). This procedure allows to estimate maturing xylem width (*w*_*maturing*_), mature xylem width (*w*_*mature*_), and increment width (*w*_*incr*_). The gray scale is unitless. It varies from 0 to 65,535 for 16 bits images used here.

Consisting of primary-walled cells, the cambium and the enlarging phloem and xylem cells have the lowest cell wall density (Figures 2, 3, 4A,B). The cell wall density increases at both sides of the cambium because of phloem and xylem cell wall differentiation (Figures 4A,B). We took advantage of this feature to define the cambium position semi-automatically by fitting a GAM and analyzing its derivative within a manually defined subset of the sample containing the cambium and the newly differentiating phloem and xylem cells (Figure 4B). Within this subset, the pointwise 95% confidence intervals of the GAM derivatives were used to detect the part where the GAM predicted values were constant (i.e., where the fitted GAM derivative values were significantly different from 0, i.e., 0 lying outside the confidence interval). The median radial position of this part was fixed as the cambium position (Figure 4B). The radial position of the previous growth ring boundary was indicated manually on the reference image (Figure 4A). It allowed to estimate the width of the increment, w_*incr*−*CT*_.

The distance from the cambium for each data point was calculated and fitted another GAM over the data spanning from the cambium (i.e., distance from the cambium = 0) to the previous growth-ring boundary. As for cambium detection, the 95% confidence intervals of the GAM derivatives allowed the identification of growth ring parts where the gray values are constant. The constant part closest to the cambium was selected as the region where the transition between maturing and mature zone occurs (Figure 4C). The radial position of the left edge of the transition region was considered as the width of the maturing xylem, w_*maturing*−*CT*_. Finally, the width of the mature zone was computed as, w_*mat*−*CT*_ = w_*incr*−*CT*_ − w_*maturing*−*CT*_. GAM fitting and derivative analysis were performed using the R packages “*mgcv*” (Wood, 2017) and “*gratia*” (Simpson, 2020). A step by step description of the gray-value profiling methodology is available in Supplementary File 1.

### Microtomy Workflow

The microcores for microtomy were dehydrated in graded ethanol series, infiltrated, and embedded in paraffin blocks and finally sliced using a rotary microtome. The thin sections were stained in a water solution of safranin and astra blue, staining lignin in red and cellulose/hemicellulose in blue, respectively. Histological sections were prepared at the Slovenian Forestry Institute (Ljubljana), following the detailed protocol of Prislan et al. (2014). Observation under light microscope allowed to distinguish between maturing and mature xylem. For each microcore, the width of the cambium, and the width of the maturing and mature xylem were measured at three positions to consider potential within-sample heterogeneity. The maturing zone was measured as the ring part between the cambium and the first mature cells. Xylem cells were considered mature when the entire cell wall was stained red and the lumen was empty of any cellular content. In order to be comparable to the HXRCT methodology, the increment width was computed as the sum of both mature and maturing xylem width and the width of the cambium divided by two. When the maturing zone was absent (i.e., for samples collected at the very end of the growing season), the mature zone was set equal to the increment width.

### Estimation of Xylogenesis Parameters and Statistical Analysis

#### Computation of Xylogenesis Critical Dates and Duration, and Growth Rate

Computation of xylogenesis phenological parameters was first performed through a “model-driven” approach, such as the fitting and derivation of the Gompertz function (e.g., Rossi et al., 2003). However, a recent methodological breakthrough suggests that a “data-driven” approach is able to capture properly the variation of xylogenesis dynamics (i.e., durations and rates) while the model-driven approach fails (Cuny et al., 2013). Preliminary analysis of the data (not shown) using a Gompertz function produced improper fitting and led us to opt for the GAM data-driven approach.

As the increase of the increment width during the growing season is monotone, a shape constrained additive model (SCAM) was used (Pya and Wood, 2015) to analyze the phenology of wood formation. SCAM was fitted using monotone increasing P-splines, Gaussian distribution, and identity link function. Circumferential growth heterogeneity was accounted for using the correction formula for cell counting data adapted to widths at the individual tree scale:

(1) $$\begin{array}{c} w_{cor,j}=w_{obs,j}*\frac{w_{prev,j}}{\frac{1}{n}\;\sum w_{prev,j}} \end{array}$$

where, *n* is the number of sampling dates, and for each *j*th sample, w_*cor,j*_ is the corrected width; w_*obs,j*_ is the observed width; w_*prev,j*_ is the observed width of the previous growth year (Rossi et al., 2003; Prislan et al., 2013).

**Figure 5:**
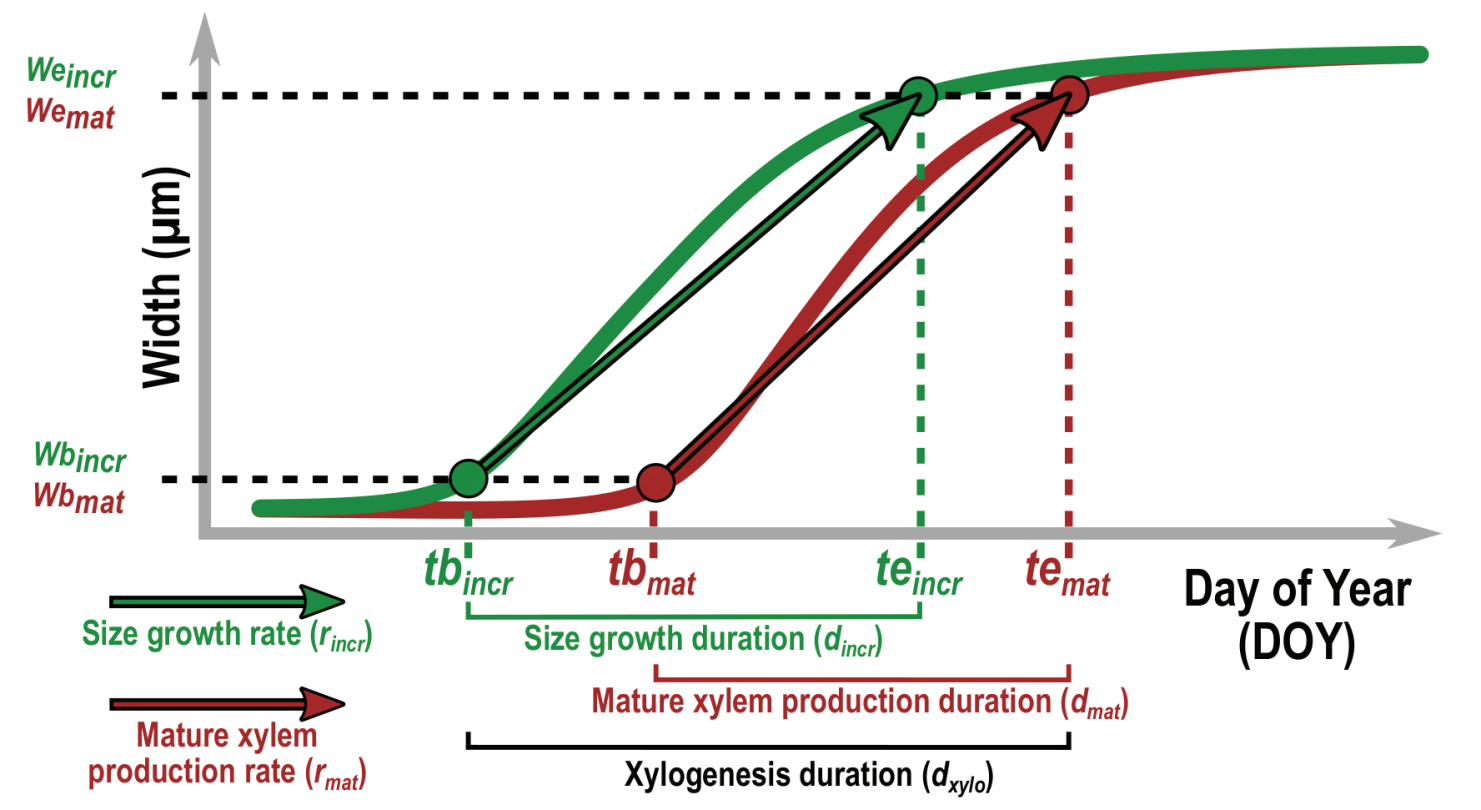
Description of the xylogenesis parameters estimated for both HXRCT and microtomy datasets. Parameters related to size growth (green) and mature xylem production (brown) are reported. The date of beginning (*tb*_*incr*_) and ending (*te*_*incr*_) of size growth are defined as the day of year for which, respectively, 5% and 95% (i.e., *wb*_*incr*_ and *wc*_*incr*_) of the increment is formed. The date of beginning (*tb*_*mat*_) and ending (*te*_*mat*_) of mature xylem production are defined as the day of year for which, respectively, 5% and 95% (i.e., *wb*_*mat*_ and *wc*_*mat*_) of the mature xylem is formed. The duration of size growth (*d*_*incr*_), mature xylem production (*d*_*mat*_) and xylogenesis (*d*_*xylo*_) are computed as the time span between *te*_*incr*_ and *tb*_*incr*_*, te*_*mat*_ and *tb*_*mat*_, and *te*_*mat*_ and *tb*_*incr*_ respectively. The average daily rate of size growth and mature xylem production are calculated as, *r*_*incr*_ = (*we*_*incr*_ − *wb*_*incr*_) / (*te*_*incr*_ − *tb*_*incr*_) and *r*_*mat*_ = (*we*_*mat*_ − *wb*_*mat*_) / (*te*_*mat*_ − *tb*_*mat*_).

First, the constrained GAM was fitted over the width of the increment to analyze the size growth dynamics at the tree level (Figure 5). The final increment width was computed as the median of the increment width predicted for the last three sampling dates (i.e., the dates for which the increment width had already stabilized), in order to account for circumferential growth heterogeneity. We defined the date of beginning (*tb*_*incr*_) and ending (*te*_*incr*_) of size growth as the day of year for which, respectively, 5% and 95% (i.e., *wb*_*incr*_ and *we*_*incr*_) of the increment is formed. The duration of size growth was computed as the time span between both dates:

(2) $$\begin{array}{r} d_{incr}=te_{incr}-\;tb_{incr} \end{array}$$

The average daily rate of size growth was calculated as:

(3) $$\begin{array}{r} r_{incr}=\left(we_{incr}-\;wb_{incr}\right)/\left(te_{incr}-\;tb_{incr}\right) \end{array}$$

The differences between the increment width predicted by GAM on the consecutive days, spanning from *tb*_*incr*_ to *te*_*incr*_, were calculated to assess the distribution of size growth daily rates.

The same procedure was applied for the width of the mature xylem in order to define the date of beginning (*tb*_*mat*_) and ending (*te*_*mat*_), the duration (*d*_*mat*_) and the average rate (*r*_*mat*_) of mature xylem production. Finally, the duration of the whole xylogenesis was defined as:

(4) $$\begin{array}{r} d_{xylo}=te_{incr}-\;tb_{incr} \end{array}$$

These parameters were estimated for both microtomy and HXRCT datasets.

#### Consistency Between HXRCT and Microtomy Measurements

The consistency between increment, mature, and maturing xylem width measured with HXRCT and microtomy was assessed using major axis regression. The value of both intercept and slope and their respective 95% confidence intervals (CI) were used to detect significant differences compared to a 1:1 regression. An intercept differing from 0 (i.e., 0 lies outside the CI of the intercept) indicates a fixed bias across the entire range, that is, the HXRCT provides greater (or lower) values than microtomy. A slope differing from 1 (i.e., 1 lies outside the CI of the slope) indicates a proportional bias, that is, the HXRCT provided progressively greater (or lower) values with increasing values of the considered measurement than microtomy. Major axes were fitted with the “*lmodel2”* package in R (Legendre, 2018).

#### Comparison of Parameters Estimated With HXRCT and Microtomy

To assess the agreement between critical dates, durations, and growth rates estimated using HXRCT and microtomy, the significant differences between the two datasets by case-based bootstrap resampling were tested. For a given species and for each parameter of interest (Figure 5), the difference between the median of the four trees based on HXRCT data on one hand, and microtomy data on the other hand was computed. The observed median difference was compared to the median reference distribution simulated under the null hypothesis (H_0_: the parameters extracted from HXRCT do not differ from the parameters extracted from microtomy). The reference distribution resulted in 10,000 resampled median differences. A two-tailed test was performed by computing the achieved significance level (ASL) as the proportion of absolute values in the reference distribution that are equal, or greater, than the observed absolute median difference. ASL is interpreted in the same way as the p value (P).

### Accounting for Biomass Production Dynamic Trough Xylem Gray Value

The ability of HRXCT to assess biomass production dynamics throughout the course of the growing season was assessed by using the gray-value information. Here, the xylem gray profile (i.e., including lumen and cell wall pixels) was preferred to the cell wall gray profile as it accounts for both the proportions of the xylem layer allocated to cell wall (biomass) and lumens (void). The sum of the gray values of each xylem gray profile was calculated. Being linearly related to the biomass accumulated in a given volume of the forming xylem ring, this variable was considered as a proxy of the biomass accumulated at the trunk base (*b*_*trunk*_) of the tree from the beginning of the growing season up to the considered sampling date. As for xylem widths, we accounted for circumferential growth heterogeneity by correcting *b*_*trunk*_ by the observed width of the previous growth year at the individual tree scale. We fitted constrained GAM over *b*_*trunk*_ to analyze the biomass production dynamics at the species level. The biomass production and the size increase were compared by analyzing the seasonal variation of both *b*_*trunk*_ and the increment width (*w*_*incr*_). For convenience, both variables were normalized (i.e., ranging between 0 and 1) using the median of *b*_*trunk*_ and *w*_*incr*_, respectively, predicted for the last three sampling dates. This provided the relative completion of each process (i.e., size growth and biomass production) and allowed to compute the time lag between biomass production and size growth on the course of the growing season. For this purpose, we computed the time lag between the relative completion of a given proportion of the final *b*_*trunk*_ and the final *w*_*incr*_ (e.g., Day of year (DOY) when a given relative completion of the final *b*_*trunk*_ is produced - DOY when a given relative completion of the final *w*_*incr*_ is produced). The time lags were computed for relative completion values ranging between 0 and 1 and every 0.01. The differences between both *b*_*trunk*_ and *w*_*incr*_ predicted by GAM on the consecutive days were also calculated. This allowed to assess the seasonal variation of size growth rate and biomass production rate and estimate their seasonal maximum.

## Results

### Seasonal Growth Pattern

**Figure 6:**
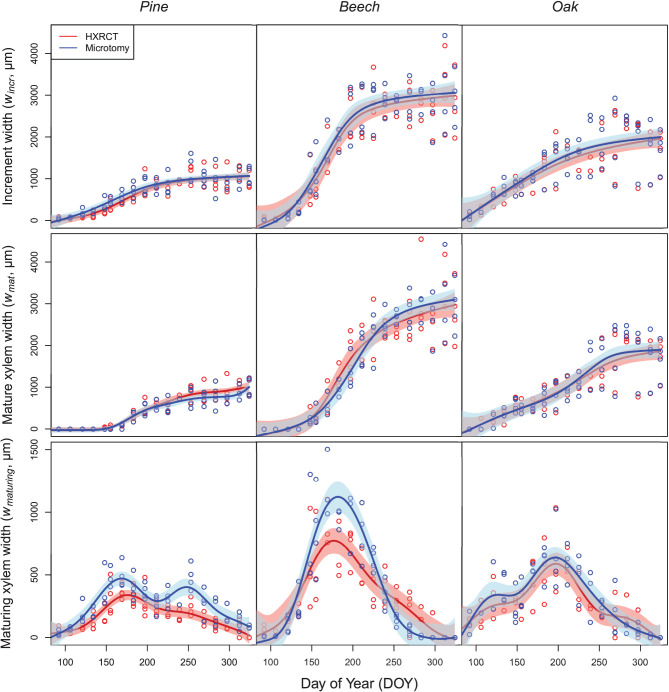
Seasonal growth pattern assessed with HXRCT (red) and microtomy (blue). Variations of increment and mature xylem were fitted with constrained GAMs (top and middle row), while maturing xylem width was fitted with unconstrained GAMs (bottom row). The shaded band represents the standard error of the fit.

The patterns of seasonal xylem growth, at the species level, derived from HXRCT and microtomy strongly overlap (Figure 6). For both pine and beech, increment and mature xylem width increased along the growing season following a typical sigmoidal curve (Figure 6). For oak, the width of the increment follows a curvilinear increase while the mature xylem width follows a bisigmoidal pattern. Major axis fits did not detect any significant bias in the estimation of increment width by HXRCT compared to microtomy (Supplementary Figure 3; Supplementary Table 1). Regarding the mature xylem width, no biases were detected for beech and oak, however, a slight slope departure from 1 for pine (Supplementary Table 1) is found. Microtomy and HRXCT described similarly the seasonal variation of maturing xylem width. For beech, the maturing xylem width followed a bell-shaped pattern while for oak and pine the pattern was bimodal, with a first peak in spring and a second peak in July for oak and in September for pine.

Overall, the estimated maturing xylem width by HXRCT is significantly lower than for microtomy for pine and beech (see Supplementary Table 1), especially during summer (Figure 6). These represent the only cases in our study when HXRCT and microtomy produce different estimates (see the third paragraph of the discussion section).

### Wood Formation Calendar

**Figure 7:**
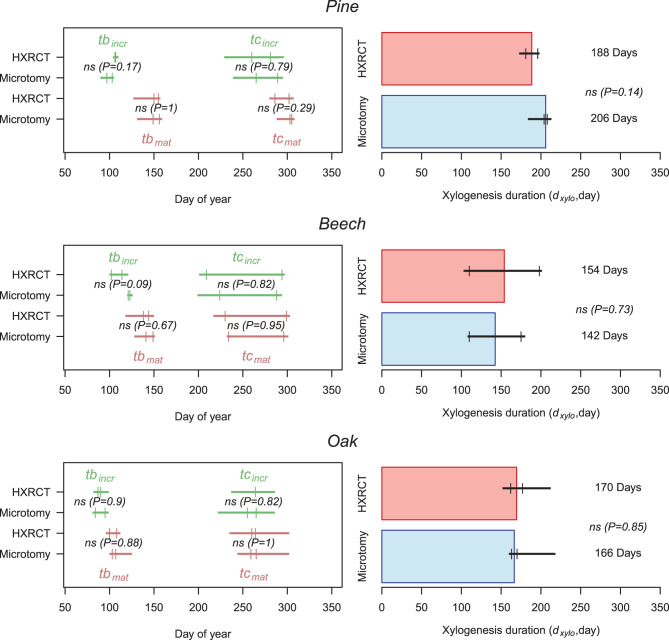
Xylogenesis calendar and duration. Left panels: Estimates of size growth beginning (*tb*_*incr*_) (i.e., beginning of xylogenesis) and size growth ending (*te*_*incr*_) (green) as well as mature xylem production beginning (*tb*_*mat*_) and ending (*te*_*mat*_) (i.e., ending of xylogenesis) (brown) are presented for HXRCT and microtomy. Green and brown lines range from minimum to maximum estimated dates, while second and third quartile are represented by short vertical lines. Right panels: Estimates of xylogenesis duration (*d*_*xylo*_) (median) for HXRCT (red) and microtomy (blue) are presented. Black lines range from minimum to maximum estimated duration, while second and third quartile are represented by short vertical lines. The significance of the bootstrap resampling test (P) assessing the difference between HXRCT and microtomy is reported.

Size growth, and therefore xylogenesis, began (*tb*_*incr*_) at the end of March in oak (one week before the first sampling date, *tb*_*incr*−*micro*_ = DOY 89, while *tb*_*incr*−*CT*_ = DOY 88, see Supplementary Table 2), while it started between mid- and end of April for pine (*tb*_*incr*−*micro*_ = DOY 100, while *tb*_*incr*−*CT*_ = DOY 106) and beech (*tb*_*incr*−*micro*_ = DOY 122, while *tb*_*incr*−*CT*_ = DOY 108, the difference is not significant). Microtomy and HXRCT gave similar estimates of *tb*_*incr*_ (Figure 7; Supplementary Table 2). Size growth was completed in mid-September for beech (*te*_*incr*−*micro*_ = DOY 256, while *te*_*incr*−*CT*_ = DOY 251), and oak (*te*_*incr*−*micro*_ = DOY 260, while *te*_*incr*−*CT*_ = DOY 264) while it lasted until the beginning of October for pine (*te*_*incr*−*micro*_ = DOY 277, while *te*_*incr*−*CT*_ = DOY 270). Again, microtomy and HXRCT provided similar estimate of *te*_*incr*_ and therefore the duration of size growth (*d*_*incr*_*)* was highly similar for both methods (*d*_*incr*−*micro*_ = DOY 177, *d*_*incr*−*CT*_ = DOY 162 for pine, *d*_*incr*−*micro*_ = DOY 134, *d*_*incr*−*CT*_ = DOY 140 for beech and *d*_*incr*−*micro*_ = DOY 163, *d*_*incr*−*CT*_ = DOY 171 for oak, the differences are not significant, see Supplementary Table 2).

The mature xylem production began in April for oak (*tb*_*mat*−*micro*_ = DOY 105, while *tb*_*mat*−*CT*_ = DOY 104), and from the end of May to beginning of June for beech (*tb*_*mat*−*micro*_ = DOY 145, while *tb*_*mat*−*CT*_ = DOY 141) and pine (*tb*_*mat*−*micro*_ = DOY 152, while *tb*_*mat*−*CT*_ = DOY 154). However, the end of mature xylem production, and therefore the end of xylogenesis, occurred by the end of September for beech (*te*_*mat*−*micro*_ = DOY 265, while *te*_*mat*−*CT*_ = DOY 264) and oak (*te*_*mat*−*micro*_ = DOY 262, while *te*_*mat*−*CT*_ = DOY 262), and by the end of October for pine (*te*_*mat*−*micro*_ = DOY 304, while *te*_*mat*−*CT*_ = DOY 294, the difference is not significant). Overall the duration of mature xylem production (*d*_*mat*_) is shorter for beech (*d*_*xylo*−*micro*_ = 142 days, while *d*_*xylo*−*CT*_ = 154 days, the difference is not significant) than for pine (*d*_*xylo*−*micro*_ = 206 days, while *d*_*xylo*−*CT*_ = 188 days, the difference is not significant) and oak (*d*_*xylo*−*micro*_ = 166 days, while *d*_*xylo*−*CT*_ = 169 days, the difference is not significant). Dates (*tb*_*mat*_*, te*_*mat*_*)* and durations (*d*_*mat*_) related to mature xylem formation as well as the duration of xylogenesis (*d*_*xylo*_) were similar across methods (Figure 7; Supplementary Table 2).

### Growth Rates

**Figure 8:**
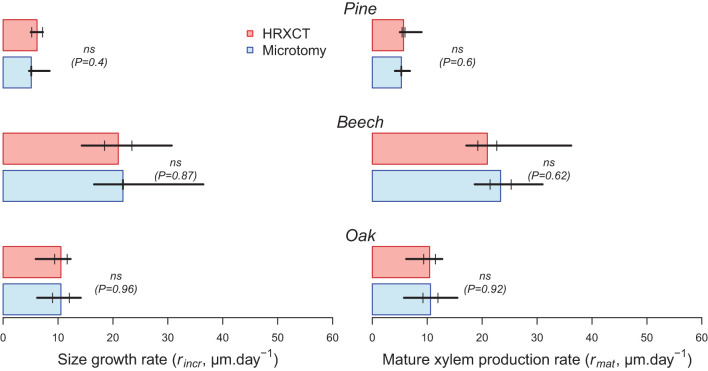
Size growth (*r*_*incr*_) and mature xylem (*r*_*mat*_) production rates (median) estimated with CT (red) and microtomy (blue). Black lines range from minimum to maximum estimated duration, while second and third quartile are represented by short vertical lines. The significance of the bootstrap resampling test (P) assessing the difference between HXRCT and microtomy is reported.

The estimated size growth rate (*r*_*incr*_) ranged from 5 to 6 μm.day^−1^ in pine to 21 to 22 μm.day^−1^ in beech. The mature xylem production rate (*r*_*mat*_) presented similar values. Overall, estimates of size growth and mature xylem production rate by microtomy and HXRCT are highly similar (Figure 8; Supplementary Table 3). Moreover, the distributions of *r*_*incr*_ and *r*_*mat*_ estimated with HXRCT and microtomy strongly overlap (Supplementary Figure 3).

### Seasonal Pattern of Biomass Production and Size Growth and Their Time Lag

**Figure 9:**
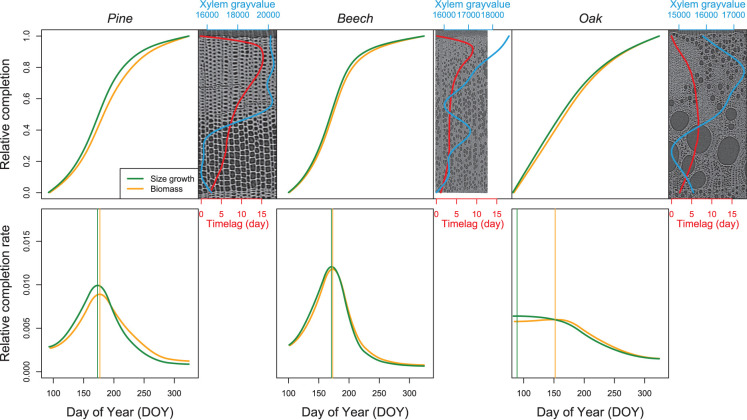
Relative completion of size growth and biomass production. The top line presents the relative completion of size growth and biomass production during the growing season as well as the time lag between size growth and biomass production (red line). For each species, a reference image and its associated xylem gray-value profile (blue line) is shown. The bottom line presents the variation of the relative completion rate, with the vertical lines highlighting the DOY at which the maximum rate occurs.

The patterns of biomass production and size growth are very similar at the species level (Figure 9). For both pine and beech, the biomass increases during the growing season following a sigmoidal curve, while biomass in oak increases curvilinearly. However, biomass production lags behind size growth. The time lag observed in respect of both size and biomass completion of the xylem ring is more important for pine (mean = 9 days, maximum=15 days) than for beech (mean = 4 days, maximum = 9 days) and oak (mean = 5 days, maximum = 7 days). For pine and beech, the time lag reaches its maximum during the latewood formation (at the end of the growing season, when 80–90% of the ring width is formed). In oak, the time lag is maximum from the end of the earlywood formation up to half of the latewood formation (between 40% and 70% of the ring formation).

The time lags observed at the peak of both size growth and biomass production rate contrast with the time lag observed in respect of both size growth and biomass completion. For both pine and beech, the relative size growth rate, as well as the biomass production rate peaked at the end of June. The difference between maximum size growth (DOY = 173) and biomass production rates (DOY = 177) was four days in pine. This difference was even smaller in beech, that is, two days (DOY = 171 vs. DOY = 173). For oak, the relative size growth rate was maximal at the end of March (DOY = 89), while the biomass production rate peaked at the beginning of June (DOY = 152), resulting in a time lag of 63 days.

## Discussion

This is the first time that xylogenesis dynamics and intra-seasonal growth rate parameters are assessed using HRXCT The development of an entire HRXCT workflow from sample preparation, to reconstructed volume analysis, allowed the estimation of relevant xylogenesis parameters (i.e., dates, durations, and rates) and seasonal time series of increment width. Moreover, the benchmarking of HRXCT with the classical microtomy proved the HRXCT approach to produce accurate estimates of xylogenesis dynamics over species with contrasted xylem anatomies.

The ability of HRXCT to encompass diverse xylem structure relies on the gray-value profiling methodology. While 2D cell morphometric analysis is not applicable on various anatomical structure, the gray-value profiling overcomes this issue by assessing the maturation state from monitoring of density at the xylem layer scale. The HRXCT methodology also proved to produce accurate estimates of actual ring width along the whole growing season ranging from ca. 30 to 5000 μm (see Supplementary Table 4). Given this result and the high resolution of HRXCT (i.e., 2.5 μm/pixel), the authors assume that HRXCT will succeed in estimating xylogenesis dynamics of slow-growing trees. Therefore, the generic and data-driven HRXCT method showed to offer a very interesting perspective to fill the knowledge gap about secondary growth phenology and intra-annual wood production over a structural and taxonomical diversity.

It is important to pinpoint that the xylem dimensions estimated with microtomy and HRXCT slightly differ in their nature. HRXCT differentiates the mature xylem from the maturing xylem on the basis of cell wall density differences. Therefore, cells in the very late stages of wood formation, for which the biomass accumulation process is almost accomplished (i.e., cells at the end of secondary wall formation and lignifying cells) or terminated (i.e., fully lignified cells but still alive) might be considered as mature cells with HRXCT. On the other hand, with microtomy such cells are considered as still maturing since the mature zone consists exclusively of fully lignified dead cells. Therefore, the slight overestimation and underestimation of the mature and the maturing zone, respectively, with HRXCT may rely on the different nature of xylem dimensions estimated with HRXCT and microtomy. However, these differences are not sufficiently important to bias the estimation of xylogenesis dynamics.

As gray scale information can be directly converted into estimates of biomass, X-ray computed tomography has been successfully used in the field of dendrochronology and biomass estimation (Bastin et al., 2015; De Mil et al., 2016). A significant step forward in the quantification of the intra-annual dynamics of carbon sequestration, for both conifers and angiosperms, relies on the calculation of the apparent density of the forming wood (i.e., the product of cell wall density and the ratio of cell wall area to xylem area) (Andrianantenaina et al., 2019). Although the apparent density approach proved to be more direct and accurate than the cell morphometric approach (Cuny et al., 2015), it still relies on physical 2D slicing of the sample. Here, it is showed that HRXCT can also be used in the field of intra-seasonal biomass production and carbon sequestration research. In fact, the sum of the xylem gray value, which is actually directly related to density (attenuation coefficients are recorded), and therefore to the biomass accumulated at the trunk base provided a very interesting insight into the capability of HRXCT to assess biomass production dynamic throughout the course of the growing season. The observed seasonal variation of the time lag between biomass production and size growth agrees with the anatomical signature of the studied species. For both pine and beech, the time lag reaches its maximum during the latewood formation, when 80–90% of the growth ring width is already achieved and thick-walled tracheids or fibers are produced. In oak, the time lag shows the largest values from the end of earlywood formation to the half of the completion of the latewood, thus between 40% and 70% of the growing ring part consists mainly of thick-walled fibers and narrow vessels. Therefore, for all species, the time lag is largest when the denser part of the growth ring is forming and suggests that wall formation and maturation of thick-walled cells require a longer period than the formation and maturation of thin-walled cells. This pattern already observed in conifers (Cuny et al., 2014) seems therefore to apply to angiosperms.

A very short time lag was found (four days) between the maximal size growth rate and the maximal biomass production rate in pine. This result strongly contrasts with the one-month time lag generally acknowledged in conifers (Cuny et al., 2015). However, Andrianantenaina et al. (2019) reported a two weeks lag between biomass production and size growth in spruce growing in north-east France. They speculated that this reduced time lag could be accounted for by the occurrence of a long summer drought during the measurement period in 2015, resulting in the shortening of the growing season and less investment in cell production. The growing season 2019 (when the samples were collected) experienced a very intense drought in late spring-early summer. Also because of the water deficit accumulated during the very dry 2018, the soil in late spring-early summer of 2019 was dry as never observed before in Belgium (Mariën et al., 2021). Therefore, wood formation in black pine might be sensitive to drought as in other conifer species (Andrianantenaina et al., 2019).

At the same site of our sampling, Dox et al. (2021) found that the end of wood formation in oak occurs earlier under drought conditions while this parameter is rather insensitive to drought in beech. In 2019, the authors found that wood formation in oak stopped earlier (DOY = 262) than in 2018 (DOY = 282 in Dox et al., 2021). As the drought in 2019 was even more pronounced than in 2018 (see above), the results corroborate the drought sensitivity of wood formation in oak.

The time lag in beech (two days) was even lower than for pine, while it was much longer in oak (63 days). However, we note that for oak, the peaks of size growth rate and of biomass production rate are much less pronounced than for the other species. During earlywood formation, from late March till early June, both size growth rate and biomass production rate were maximal and rather constant in oak. We believe that the large time lag in oak results from the ring-porous structure of its wood, which implies a fast size growth, due to the enlargement of the vessels, at the beginning of the growing season. Given the uniqueness of such parameters computed for angiosperms, any comparison with previous studies is not possible.

We believe that further development of HRXCT will open the way for more insight into biomass production in angiosperms.

Therefore, we expect that the new HRXCT workflow presented here will be the foundation for major breakthroughs also in the field of intra-seasonal biomass production and carbon sequestration research. Together with a proper conversion of gray value into wood density and species-specific allometric relationships, HXRCT has the clear potential to provide a better quantification of intra-annual dynamics of biomass production and carbon sequestration through high-resolution 3D mapping of carbon within the forming growth ring and among different tree species.

HXRCT is a growing and wide spreading technology in the field of tree- and wood sciences. Both forthcoming technological improvements of scanning devices and protocol optimization (batch preparation and actual scanning of samples), as well as increased computer capacity, will offer potential for the further development of a high-throughput analysis of xylogenesis with HXRCT. Such development will allow to (1) cover a broad range of biomes and climates by increasing drastically the number of sampled individuals and to (2) integrate different tree components (i.e., root, stem, and branches) that may present different phenological patterns and react differently to similar climatic conditions. Thus, the HRXCT approach, developed here, offers a promising avenue to investigate seasonal plant-climate growth relationships spanning a wide range of wood structures, species, biomes, and climatic conditions, and to generate a substantial amount of data that could trigger a new generation of vegetations models fully considering the seasonal wood growth dynamics.

## Data Availability Statement

The raw data supporting the conclusions of this article will be made available by the authors on request, without undue reservation.

## Author Contributions

RL, JVdB, and MC designed the study. BM, MC, and RL collected the samples. JG and PP processed the sample for microtomy. RL and JG conducted the analysis of the histologic slices. RL conducted the X-ray computed tomography analysis and the analysis of the data. RL, JVdB, MC, and HB wrote the first draft of the manuscript. RL, JVdB, MC, and HB wrote the revised manuscript. BM, JG, and PP made significant contributions to the manuscript. All authors gave final approval for publication.

## Conflict of Interest

The authors declare that the research was conducted in the absence of any commercial or financial relationships that could be construed as a potential conflict of interest.

## Publisher's Note

All claims expressed in this article are solely those of the authors and do not necessarily represent those of their affiliated organizations, or those of the publisher, the editors and the reviewers. Any product that may be evaluated in this article, or claim that may be made by its manufacturer, is not guaranteed or endorsed by the publisher.
