# Supplementary material for: High-Resolution X-Ray Computed Tomography: A New Workflow for the Analysis of Xylogenesis and Intra-Seasonal Wood Biomass Production
Source: Front Plant Sci. 2021 Aug 6;12:698640. doi: 10.3389/fpls.2021.698640 (PMC8377475; doi:10.3389/fpls.2021.698640)
Supplement: Supplementary file 1 [file Data_Sheet_1.zip › Supplementary Figure 1.PDF]

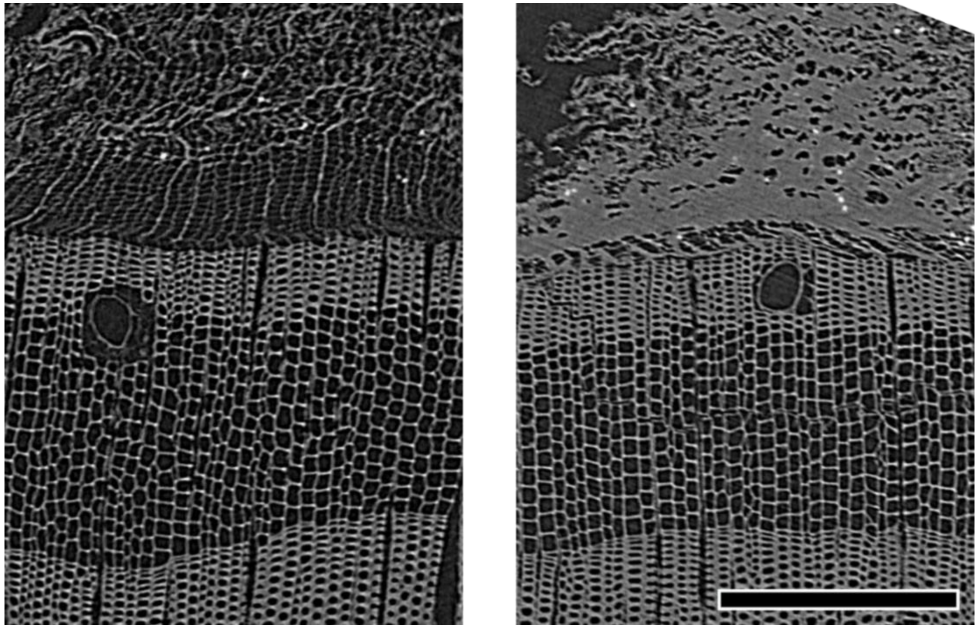

Supplementary Figure 1. Comparison between a critical point dried (left) and an air dried microcore (right). The microcores were collected at the same date and on the same individual. Scale bar 500 µm.
