# Supplementary material for: High-Resolution X-Ray Computed Tomography: A New Workflow for the Analysis of Xylogenesis and Intra-Seasonal Wood Biomass Production
Source: Front Plant Sci. 2021 Aug 6;12:698640. doi: 10.3389/fpls.2021.698640 (PMC8377475; doi:10.3389/fpls.2021.698640)
Supplement: Supplementary file 1 [file Data_Sheet_1.zip › Supplementary Figure 2.PDF]

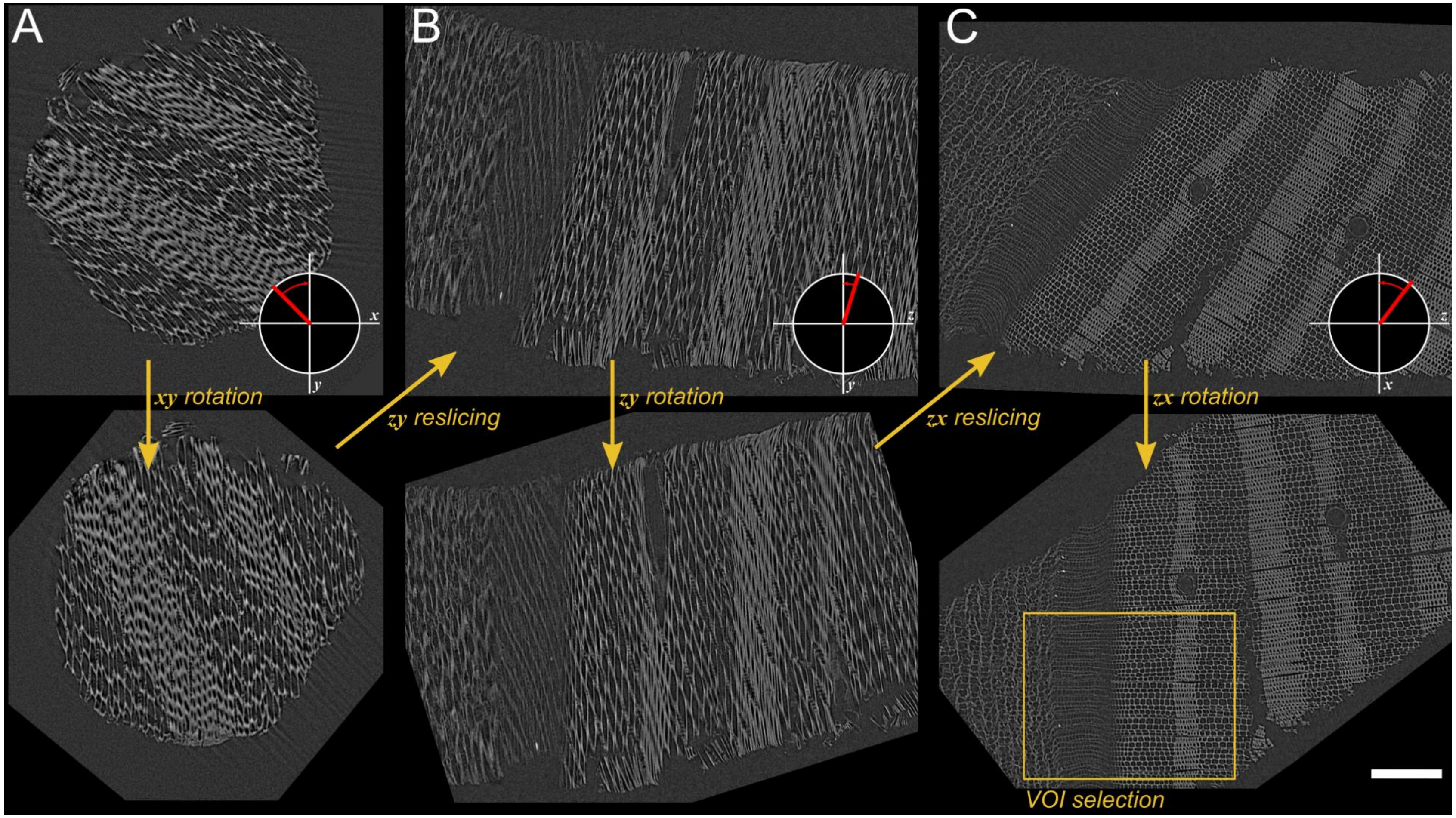

Supplementary Figure 2. Description of the re-orientation process of the sample. (A) Orientation of the fibers parallel to the edge of the volume (correction of sample positioning during scaning), (B) orientation of the cambium (or growth ring boundary) parallel to the volume edge in the longitudinal plane and (C) orientation of the cambium (or growth ring boundary) parallel to the volume edge in the transverse plane and selection of the volume of interest (VOI). Scale bar = 500µm
