# Supplementary material for: High-Resolution X-Ray Computed Tomography: A New Workflow for the Analysis of Xylogenesis and Intra-Seasonal Wood Biomass Production
Source: Front Plant Sci. 2021 Aug 6;12:698640. doi: 10.3389/fpls.2021.698640 (PMC8377475; doi:10.3389/fpls.2021.698640)
Supplement: Supplementary file 1 [file Data_Sheet_1.zip › Supplementary Figure 3.DOCX]

|  |
| --- |
| Supplementary Figure 3. Major axis (MA) regression between xylem dimensions measured with HXRCT and microtomy for thee species studied. Species are highlighted by different colors. The colored solid lines are the major axis fit for each species while the y=x line is represented by the gray solid line. |
