# Supplementary material for: High-Resolution X-Ray Computed Tomography: A New Workflow for the Analysis of Xylogenesis and Intra-Seasonal Wood Biomass Production
Source: Front Plant Sci. 2021 Aug 6;12:698640. doi: 10.3389/fpls.2021.698640 (PMC8377475; doi:10.3389/fpls.2021.698640)
Supplement: Supplementary file 1 [file Data_Sheet_1.zip › Supplementary File 1.DOCX]

**Supplementary File 1. Step by step description of the gray-value profiling methodology**

Following is an in-depth and illustrated description of the different steps of the gray-value profiling methodology. The implementation was done in the statistical software R. The example involves a beech core collected the July 16^th^ 2019.

The methodology relies on the analysis of the cell-wall gray-value profile by Generalized Additive Models (GAM). GAMs were preferred because they offer the possibility to (1) describe accurately the diversity of non-linear trends, (2) derive the fitted function and assess the significance of gray-value changes along the xylem increment, and finally, (3) are not computationally intensive. The packages ‘*mgcv*’ (Wood, 2017) and ‘*gratia*’ (Simpson, 2020) were used to fit each Generalized Additive Model and to assess the derivative of each fit. Alls GAMs were fitted with Gaussian distribution and identity link function. The goodness of each fit was assessed visually.

Step 1: The cell-wall gray-value profile of the sample is plotted on the transversal reference image, with each data point (yellow triangles) corresponding to the average gray value of the corresponding tangential slice.

Step2: The subset of the sample containing the cambium is demarcated manually (red vertical dashed lines). This subset contains the newly differentiating xylem and phloem cells, as well as the cambium initials.

Step3: A generalized additive model (GAM) is fitted (red solid curve) on the subset demarcated in step2.

Step4: The derivative of the GAM function fitted in step3 is computed. The part where the derivative does not significantly differ from 0 is identified (blue dots) (i.e. where 0 lies inside the pointwise 95% confidence interval of the GAM derivative). This part is considered as the region containing the cambium.

Step5: The bounding edges of the part containing the cambium are identified automatically (blue vertical dashed lines).

Step6: The cambium position (vertical green line) is calculated as the median position of the bounding edges identified in step5.

Step7: The boundary between the current and the previous growth ring (orange vertical line) is manually identified on the reference image.

Step8: The identification of the cambium position allows to calculate the distance from the cambium for each data point (note the change of the X-axis between step7 and step8) and to estimate the increment width (w_incr_).

Step9: Another GAM is fitted (red solid curve) over the data ranging from the cambium to the previous growth-ring boundary.

Step10: The derivative of the GAM function fitted in step9 is computed. The pointwise 95% confidence intervals of the GAM derivative allow the identification of growth ring parts where the gray-values are constant (blue dots).

Step11: The constant part which is the closest to the cambium is identified as the region where the transition between maturing and mature zone occurs. Its left edge (blue vertical line) is considered as the width of maturing xylem width (w_maturing_).

Step12: Knowing w_incr_ and w_maturing_, the mature xylem width (w_mat_) is computed. In this example, w_incr_=2462 µm, w_maturing_=635µm and w_mat_=1827 µm
