# Supplementary material for: High-Resolution X-Ray Computed Tomography: A New Workflow for the Analysis of Xylogenesis and Intra-Seasonal Wood Biomass Production
Source: Front Plant Sci. 2021 Aug 6;12:698640. doi: 10.3389/fpls.2021.698640 (PMC8377475; doi:10.3389/fpls.2021.698640)
Supplement: Supplementary file 1 [file Data_Sheet_1.zip › Supplementary Table 1.DOCX]

Supplementary Table 1. Major axis (MA) regression between xylem dimensions measured with HXRCT and microtomy for the species studied. Regression coefficients and 95% confidence intervals are presented. The coefficient of determination (R²) of the ordinary least square regression is also presented.

| Regression | Species | Intercept [CI] | Slope [CI] | R² |
| --- | --- | --- | --- | --- |
| Increment width | *Pinus nigra* | -48.9 [-91.29; -8.99] | 1 [0.94;1.06] | 0.95 |
|  | *Fagus sylvatica* | -56.92 [-146.05; 28.76] | 1.01 [0.98; 1.06] | 0.98 |
|  | *Quercus robur* | -18.87[-80.14; 39.81] | 1. [0.96; 1.04] | 0.98 |
| Mature xylem width | *Pinus nigra* | 0.82 [-24.23; 24.51] | 1.09 [1.03; 1.15] | 0.96 |
|  | *Fagus sylvatica* | 61.75 [-41.3; 159.21] | 0.99 [0.94; 1.05] | 0.96 |
|  | *Quercus robur* | 36.2[-19.71; 89.43] | 0.97 [0.92; 1.02] | 0.97 |
| Maturing xylem width | *Pinus nigra* | 2.02 [-27.85; 28.97] | 0.64 [0.53; 0.76] | 0.68 |
|  | *Fagus sylvatica* | 86.54 [53.17; 117.66] | 0.62 [0.54; 0.7] | 0.82 |
|  | *Quercus robur* | 18.65 [-18.27; 51.36] | 0.86 [0.75; 0.99] | 0.8 |
