# Supplementary material for: High-Resolution X-Ray Computed Tomography: A New Workflow for the Analysis of Xylogenesis and Intra-Seasonal Wood Biomass Production
Source: Front Plant Sci. 2021 Aug 6;12:698640. doi: 10.3389/fpls.2021.698640 (PMC8377475; doi:10.3389/fpls.2021.698640)
Supplement: Supplementary file 1 [file Data_Sheet_1.zip › Supplementary Table 3.DOCX]

**Supplementary Table 3. Size and mature xylem growth rate estimated with microtomy and HRXCT.** The median and the minimum and maximum values are presented. For each species and parameter, the significance of the bootstrap resampling test (P) assessing the difference between HXRCT and microtomy is reported.

|  |  | *Size growth rate (r_incr_)* [min ; max] |  | *Mature xylem production rate (r_mat_)* [min ; max] |  |
| --- | --- | --- | --- | --- | --- |
| *Pine* | *Microtomy* | 5.1 [4.7 ; 8.5] | *P = 0.4* | 5.3 [4.1 ; 6.9] | *P = 0.6* |
| *Pine* | *HXRCT* | 6.2 [5 ; 7.2] |  | 5.7 [5 ; 9] |  |
| *Beech* | *Microtomy* | 21.8 [16.6 ; 36.5] | *P = 0.87* | 23.4 [18.7 ; 31] | *P = 0.62* |
| *Beech* | *HXRCT* | 21 [14.3 ; 30.7] |  | 20.9 [17.1 ; 36.2] |  |
| *Oak* | *Microtomy* | 10.5 [6.2 ; 14.1] | *P = 0.96* | 10.6 [5.8 ; 15.5] | *P = 0.92* |
| *Oak* | *HXRCT* | 10.5 [5.9 ; 12.3] |  | 10.4 [6.2 ; 12.7] |  |
