# Supplementary material for: High-Resolution X-Ray Computed Tomography: A New Workflow for the Analysis of Xylogenesis and Intra-Seasonal Wood Biomass Production
Source: Front Plant Sci. 2021 Aug 6;12:698640. doi: 10.3389/fpls.2021.698640 (PMC8377475; doi:10.3389/fpls.2021.698640)
Supplement: Supplementary file 1 [file Data_Sheet_1.zip › Supplementary Table 4.DOCX]

**Supplementary Table 4. Summary of the increment width measured with microtomy and HRXCT.** The averages of the increment width 2019 and their range (min-max) are presented. The averages of final increment width 2019 and 2018 together with their standard deviation (sd) are also presented.

|  | Increment width 2019 (min-max) (µm) | | Final increment width 2019 (sd) (µm) | | Final increment width 2018 (sd) (µm) | |
| --- | --- | --- | --- | --- | --- | --- |
|  | *Microtomy* | *HRXCT* | *Microtomy* | *HRXCT* | *Microtomy* | *HRXCT* |
| Pine | 703 (30-1896) | 639 (31-1773) | 1167 (319) | 1215 (349) | 1282 (541) | 1254 (530) |
| Beech | 2012 (7-4914) | 2115 (32-5029) | 3104 (891) | 3156 (965) | 2297 (772) | 2290 (788) |
| Oak | 1398 (28-3047) | 1274 (63-2829) | 1793 (582) | 1792 (580) | 1449 (404) | 1489(518) |
